# Artificial dielectric beam-scanning prism for the terahertz region

**DOI:** 10.1038/s41598-023-41046-z

**Published:** 2023-08-23

**Authors:** Karl Strecker, Matthew Otto, Masaya Nagai, John F. O’Hara, Rajind Mendis

**Affiliations:** 1 School of Electrical and Computer Engineering, Oklahoma State University, Stillwater, OK 74078 USA; 2 Graduate School of Engineering Science, Osaka University, Toyonaka, Osaka 560-8531 Japan; 3 Riverside Research, Open Innovation Center, Beavercreek, OH 45431 USA

**Keywords:** Electrical and electronic engineering, Optical materials and structures

## Abstract

We design and fabricate an artificial dielectric prism that can steer a terahertz beam in space and experimentally investigate its behavior. The artificial dielectric medium consists of a uniformly spaced stack of metal plates, electromagnetically equivalent to an array of parallel-plate waveguides operating in tandem. At an operating frequency of 0.3 THz, we observe a maximum beam deflection of 29°, limited by the precision of the available spacers. Spring-loading the spacers between the plates allow us to scan the beam continuously and dynamically over a range of 5°. The measured beam intensity maps at the input and output of the device reveal very good Gaussian beam quality and an estimated power efficiency of 71%. As a possible real-world application, we integrate the prism into the path of a free-space terahertz communication link and demonstrate unimpaired performance.

## Introduction

Future concepts of wireless communications and sensing increasingly involve the terahertz frequency band (0.1–10 THz). For example, terahertz or greater frequencies are an essential element of next-generation (6G) communications, wherein bandwidths of ~ 100 GHz become critical to support the terabit-per-second data rate expectations^1^. To achieve practical terahertz wireless systems, numerous research studies have addressed the challenges of wave absorption, scattering, digital signal processing, networking, security, media access control, transceiver development, and more^2,3^. Another notable and fundamental challenge is free-space path loss (FSPL). Owing to its scaling with the square of frequency, FSPL becomes distinctly worse in the terahertz regime than in lower frequency bands. This greatly impacts both sensing (e.g. radar) and communication systems since it requires terahertz beams to be highly directive to achieve practically significant propagation distances. Consequently, it spawns new challenges involving beam pointing, jitter, and turbulence. Active beam scanning (or steering) is the proffered solution, inspiring myriad approaches including phased arrays, reconfigurable diffractive or reflective surfaces, and dispersive structures^4^. Some recent examples include a complex optical system utilizing mirrors^5^, two based on sophisticated metasurfaces^6,7^, one based on a Luneburg lens^8^, one based on phased arrays^9^, one based on a diffraction grating^10^, and one based on a 3D-printed prism^11,12^. Many of these perform admirably, although they can suffer from low efficiency, poor beam quality, high complexity, or limited bandwidth, particularly if they offer dynamic control. In future applications like next-generation wireless communications, it will be important that wave control devices carefully avoid poor efficiency^13^, beam anomalies (e.g. squint), and waveform reshaping due to loss and temporal dispersion^14^.

Among the solutions involving dispersive structures, artificial dielectrics (ADs) become very attractive. Artificial dielectrics are man-made media that mimic the properties of naturally occurring dielectric media, or even manifest properties that cannot generally appear in nature^15^. For example, the refractive index, which usually has a value greater than unity, can have a value less than unity in an AD. Recent studies^16,17^ have shown that ADs provide powerful avenues for terahertz wave control, analogous to metamaterials, but with practical advantages like greatly reduced absorption losses and significantly reduced fabrication complexity. These properties are manifested in new AD-based terahertz isolator and beamsplitter designs whose specifications rival even mature optical-wave devices^17^.

Exploiting this AD concept, here, we design and fabricate a dynamic beam-scanning prism for the terahertz region and experimentally investigate its behavior. Compared to most beam scanners, our AD device is much simpler, resulting in superior beam quality, higher power efficiency, and low temporal dispersion. We envision that this work will be important for advancing terahertz wireless communications, imaging, and remote sensing. In the case of wireless communications, terahertz waves are increasingly poised for adoption in point-to-point links, such as backhaul applications^18^. In such scenarios, the ability to optimally steer a transmitter beam to a receiver location is paramount, especially if the receiver is mobile or the channel is affected by jitter.

## Design and fabrication

**Figure 1:**
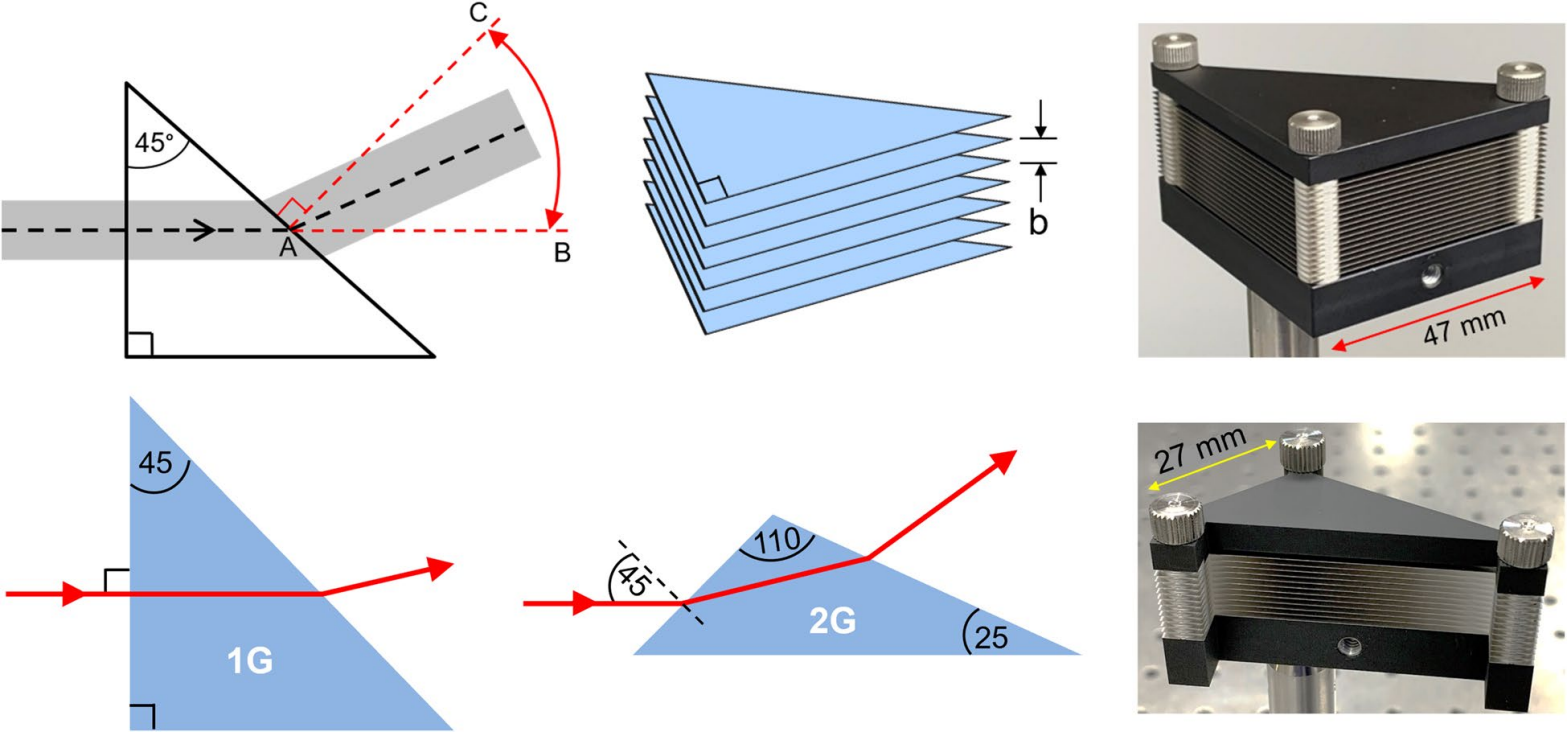
Artificial-dielectric prism. The illustrations in the top row (from left to right) show the schematic, an exaggerated 3D rendering, and a photograph of the fabricated prism. The gray area in the schematic denotes the terahertz beam. The spacers are not shown in the rendering for clarity. The illustrations in the bottom row (from left to right) show the ray-optics analyses for the first-generation and second-generation prisms, and a photograph of the fabricated second-generation prism.

The design and fabrication of the beam-scanning prism is illustrated in the top row of Fig. 1, where the AD medium consists of a stack of metal plates. Each plate has an isosceles right-triangular shape and is fabricated using 100 µm thick stainless steel. The prism is assembled with a uniform separation *b* between the plates using stainless-steel spacers. Both the plates and spacers are fabricated by a process of chemical etching to avoid any strain or burring, and also to maintain their flatness. The device is assembled by stacking the plates and spacers alternating along three mounting-rods positioned at the corners of the triangle. Once assembled, this stacked-plate arrangement has an aperture with a height that is sufficiently large to accommodate an input beam with a 1/*e* Gaussian field diameter of 10 mm.

As shown in the schematic in Fig. 1, a terahertz beam that enters the prism at normal incidence will propagate through the prism while experiencing an effective refractive index given by *n* = [1 − (*c*/2*bf*)^2^]^0.5^, where *c* is the free-space velocity and *f* is the operating frequency^1,19^. Note that based on this theoretical expression, the refractive index can have any value between zero and unity by a suitable choice of *b*. At the exit face, the beam encounters an index mismatch, transitioning from the AD medium (*n* < 1) to free space (*n* = 1). Therefore, similar to a glass prism that can bend a beam of light, this AD prism will bend a terahertz beam as it exits the prism in accordance with Snell’s law. When *n* is close to unity, the beam will exit the prism along AB, and when *n* is close to zero, the beam will exit along AC. Thus, simply by varying the plate spacing (i.e., compressing and expanding the plate assembly), it is possible to steer the output beam through a total angle of 45°.

**Figure 2:**
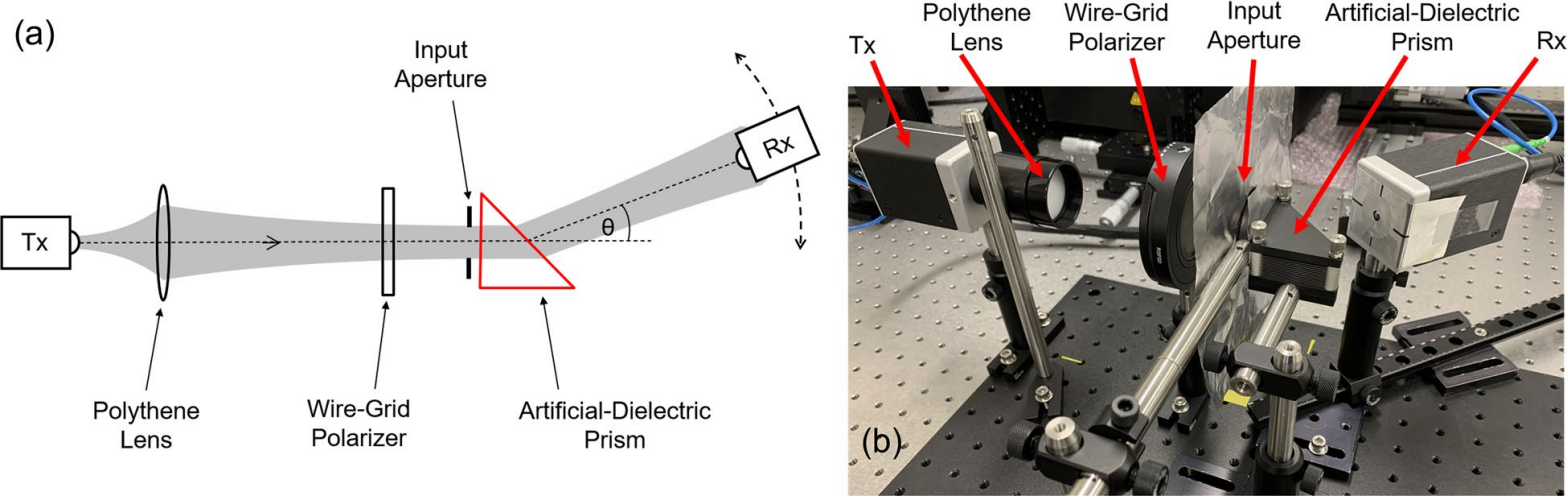
Experimental setup. (**a**) Schematic diagram. (**b**) Photograph of actual setup.

Experimental studies were carried out on a test device [shown top right in Fig. 1] using a fiber-coupled pulsed terahertz system in a transmission configuration. Figure 2 shows the schematic diagram of the experimental setup along with a photograph of the actual setup. Similar to the schematic shown in Fig. 1, a well-collimated terahertz beam was coupled perpendicular to the input face of the prism. The input electric field was polarized parallel to the plates to excite the lowest-order transverse-electric (TE_1_) mode of the parallel-plate waveguides (PPWGs) that constitute the plate stack^20^. A wire-grid polarizer was used to enhance the purity of the linear polarization of the input beam. While this configuration does not strictly forbid excitation of some higher-order modes, from a practical standpoint, it has been verified that only the TE_1_ mode is significantly excited^20^. The input face of the prism was located at the focus of a polythene lens (having a focal length of 15 cm) affixed to the transmitter head. A 12 mm diameter aperture (with an aluminum-sheet background) was positioned at the input to define the beam axis and prevent any energy leakage around the prism. During the experiment, the output signal was detected at various angular positions by scanning the receiver in the azimuthal plane along the BC arc, equidistant from A. Due to the finite (6 mm diameter) aperture of the receiver, only a part of the radiated broadband terahertz beam was collected at each angular position.

## Results and discussion

**Figure 3:**
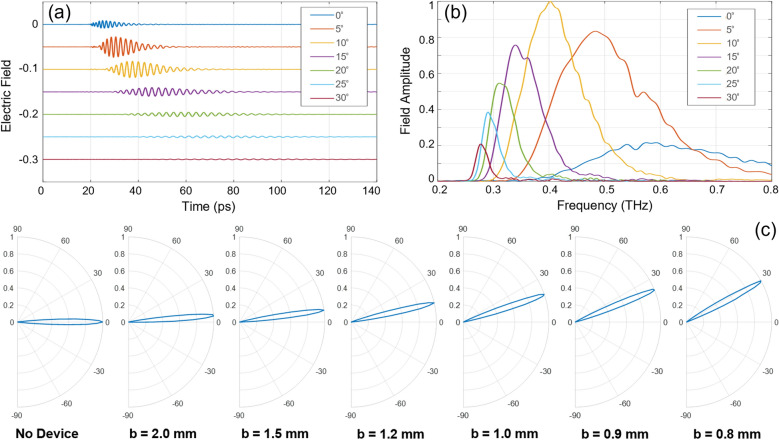
Experimental results. (**a**) Detected signals at various angular positions for *b* = 0.8 mm. (**b**) Corresponding amplitude spectra. (**c**) Measured azimuthal polar plots of the output beam at 0.3 THz for plate separations decreasing from 2 to 0.8 mm compared to the No-Device case.

Figures 3(a) and (b) respectively show a representative set of detected temporal signals and their Fourier-transformed amplitude spectra for *b* = 0.8 mm. The signal waveforms display a negative chirp (high frequencies arriving earlier in time) characteristic of the expected TE_1_-mode propagation behavior^20^. As the detection angle increases, there is a red shifting of the spectrum, implying that the lower frequencies tend to refract more for a given plate separation.

Similarly, for any single frequency, the azimuthal deflection angle varies with plate separation. By extracting individual spectral amplitudes from the broadband measured data, we can construct the azimuthal polar plots of the output beam for various plate separations. These results are given in Fig. 3(c) for 0.3 THz, the design frequency. Each of these plots was obtained by measuring time-domain scans for 66 azimuthal positions in 1° increments to generate the polar radiation pattern. The “No Device” case defines the input beam axis at 0°, and this case is compared to the situations when the prism was assembled with progressively decreasing plate separations. We observe a clear steering of the output beam, where the refraction angle increases as *b* decreases. This interdependence is consistent with the theoretical expression above, since a decreasing *b* decreases *n*, which in turn increases the scan angle due to a higher index mismatch. Furthermore, based on these experimental polar plots, we estimate a half-power-half-angle of 4° for all of the azimuthal beams. The input aperture of the device is sufficiently larger than the input beam diameter, and thus the device has no impact on the output beam spreading which is essentially the free-space (No-Device) spreading of the input beam that is also 4°.

Although these *static-test* results were promising, the above-mentioned device had a few shortcomings. For example, since the flatness of the plates played an important role in determining the equivalent electromagnetic properties of the device, the performance degraded when we moved onto smaller plate separations. This was because irregularities in the plate spacing became appreciable at smaller values of the separation, causing the observed deflection to deviate significantly from the theoretically expected value. Therefore, to overcome these issues, we designed and fabricated an optimized prism.

The development of this second-generation prism is illustrated in the bottom row of Fig. 1. In this new design, we utilized both the entrance and exit faces to bend the beam, unlike the first design where bending occurred only at the exit face. Thus, here, the beam enters the prism at 45°, and is no longer at normal incidence as in the original design. We performed a design optimization process via ray-optics as shown in Fig. 1, using both direct analysis and numerical simulations. In the new design, at *b* = 0.8 mm, the theoretical deflection angle for a 0.3 THz beam is 31°, whereas it is only 11° in the original design. Thus, this new geometry allowed us to realize a larger deflection angle for a given plate separation. We also made the new design more compact by reducing the overall span of the individual plates, thereby minimizing the flatness variance. The measured azimuthal polar plots shown in Fig. 3(c) are the ones obtained using this second-generation prism.

**Figure 4:**
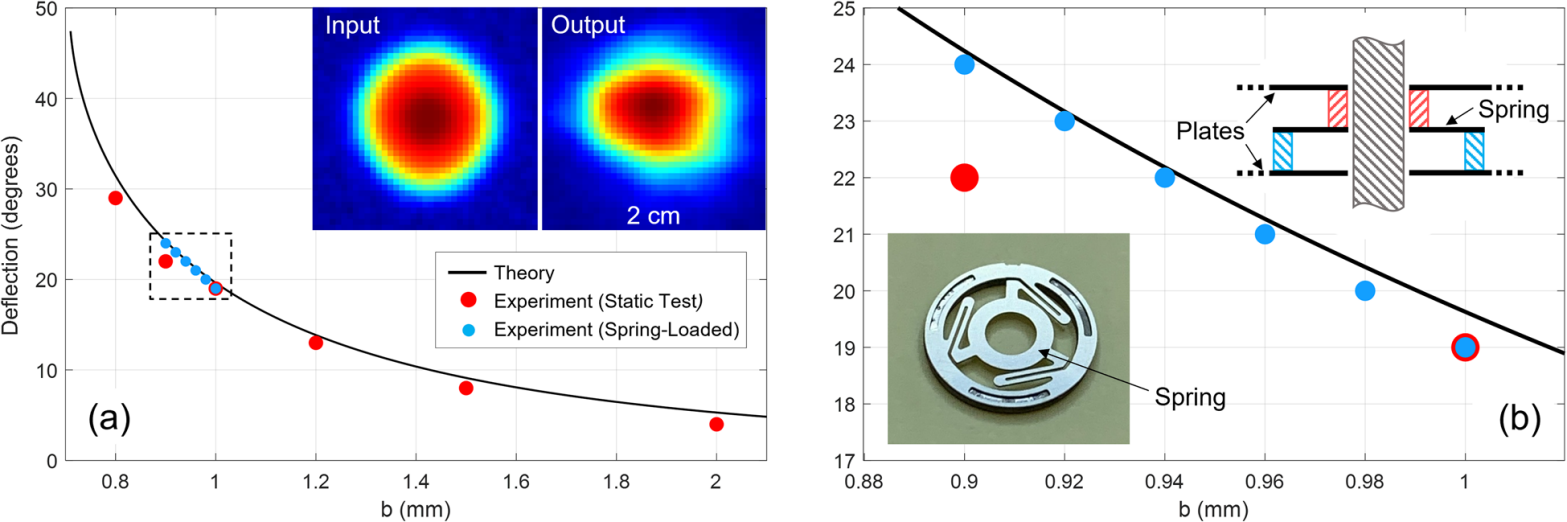
(**a**) Beam deflection versus the plate separation *b* for the second-generation prism. The insets give the measured input and output beam intensity maps at 0.3 THz. (**b**) Closeup view of the dashed rectangular region of the plot in (**a**). The bottom inset shows a photograph of the ortho-planar spring. The top inset shows a cross-section illustrating the rod and spring mechanism. The red and blue hashed areas denote two washers attached to the ortho-planar spring.

The experimental deflection angle observed for this second-generation prism is plotted in Fig. 4(a) as the red dots, compared to the theoretical prediction given by the black curve. This comparison shows very good agreement between experiment and theory, with a maximum experimental deflection of 29°, limited only by the precision of the available spacers. In fact, the theory predicts deflection angles beyond 45°, which should be practically feasible given the close match between theory and experiment. Furthermore, the insets in Fig. 4(a) illustrate the measured 2D beam intensity maps at the input (left panel) and output (right panel) of the prism for *b* = 1.2 mm. These maps were generated by raster scanning the receiver (affixed with a 1-mm aperture to improve the spatial resolution) in the vertical plane in a 2 cm square grid with a 0.5 mm step size. These maps were generated at 0.3 THz and prove that both the input and output beams have Gaussian profiles (albeit with some minor asymmetry in the output beam) and thus good beam quality. These intensity maps also allow us to estimate the overall power efficiency of the device, revealing an efficiency of 71% for the prism with a plate separation of 1.2 mm.

As an extension to the static test, in a separate experiment, we incorporated *dynamic* scanning to the prism by spring-loading the precision spacers that are sandwiched between the plates. To continuously vary the plate separation, mechanical pressure was externally applied to the plate assembly. These experimental results are also plotted in Fig. 4(a) as the blue dots, with a close-up view of the region-of-interest shown in Fig. 4(b). The insets in Fig. 4(b) illustrate the spring mechanism based on what are known as ortho-planar springs^21^. These are high-precision springs that are cut out of planar metal sheets, in this case 100 µm thick stainless-steel. The top inset shows a cross-section of the spring and the mounting rod, while the bottom inset shows a photograph of the actual ortho-planar spring. The spring action is achieved by mechanically displacing the inner circle of the spring from the outer circle.

The outer diameter of each ortho-planar spring is only 6 mm, exactly the same size as the diameter of the fixed spacers used in the static test. Therefore, replacing the fixed spacers with the ortho-planar springs does not affect the lateral dimensions of the air gaps through which the beam propagates, and thus, causes no interference to the beam. Furthermore, as seen in the photographs of Fig. 1, the prism consists of a bottom plate and a top plate made of anodized aluminum. In the experiment, the bottom plate is fixed in space and carries three (vertical) mounting rods at the corners of the triangle to support the plate assembly. Each stainless-steel plate (plus the top plate) has three mounting holes that precisely align with these rods. In the dynamic test, the three screws that secure the top aluminum plate are removed, such that this plate can freely slide along the three mounting rods. Then, with the ortho-planar springs installed between the plates, a mechanical displacement is applied to this top plate via a micrometer actuator arm affixed to this plate, while making sure that the axis of motion is precisely vertical. This ensures that the mechanical displacement is equally distributed to the three corners, thus simultaneously compressing and de-compressing the entire plate assembly in unison.

As shown by the blue dots in Fig. 4(b), by using this structure, we successfully demonstrate precisely controlled deflection of the terahertz beam in 1° increments by continuously varying the plate separation. Thus, the ability to continuously scan the beam in a dynamic fashion was demonstrated, which would be required in a fully functional device. In this experiment, the displacement was limited to 100 µm (*b* decreasing from 1.0 to 0.9 mm), to ensure that the spring operated within its elastic regime. This demonstration also confirmed that the action of all the springs was uniform, such that the plate spacings also remained uniform during the compression (and expansion) of the plate assembly. Had it not been uniform during the dynamic operation, the experimental polar plots (that were used to extract the beam deflection) would have shown signs of side-lobes in the radiation patterns. This was not the case, and the shapes of the derived polar plots were almost identical to the ones seen in Fig. 3(c), confirming a clean and uniform operation.

## Real-world demonstration

**Figure 5:**
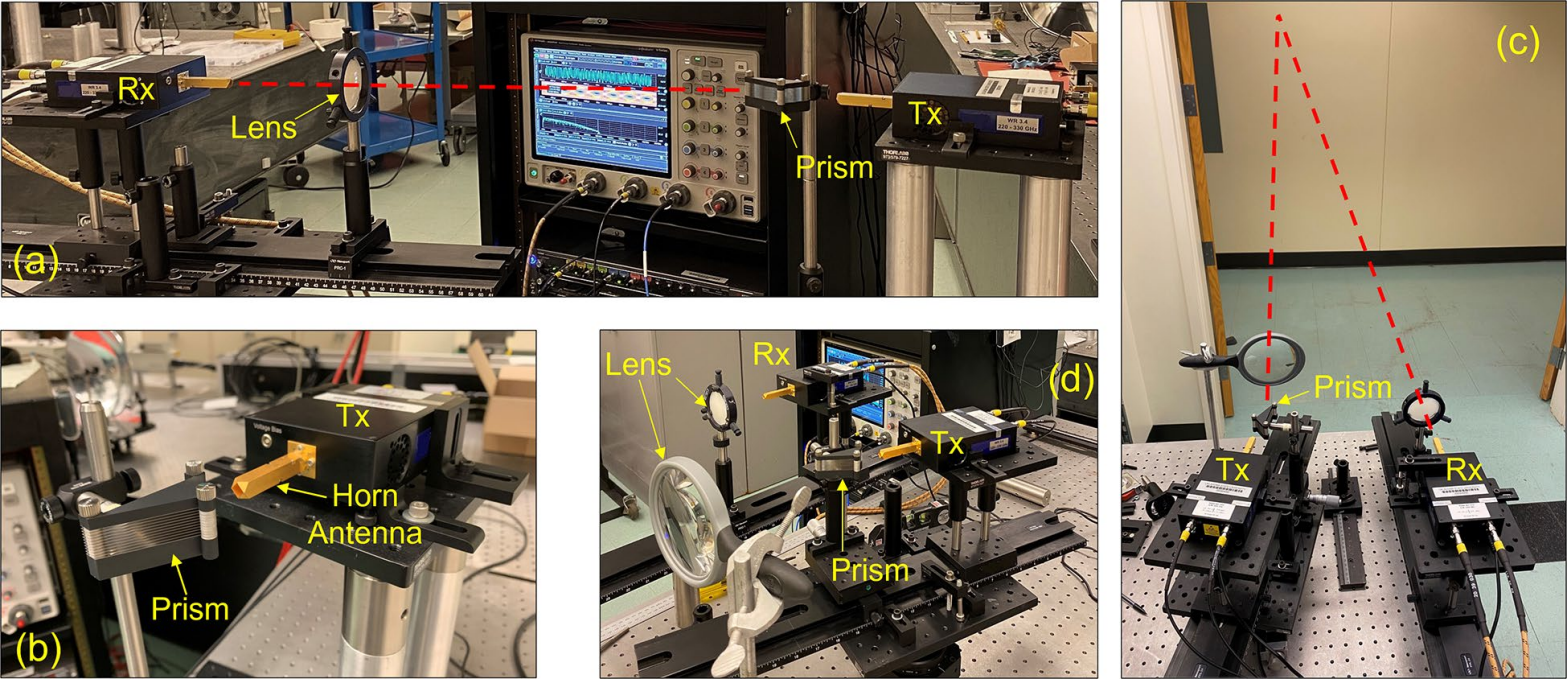
Terahertz communication demonstrations. (**a**) Communication link with a 0.5 m line-of-sight path. (**b**) Closeup view of the prism and the diagonal horn antenna attached to the transmitter. (**c**) Communication link with a 7.5 m reflection path, where the terahertz beam reflects off finished drywall. (**d**) Closeup view of the 7.5 m link from the opposite side showing the prism, transmitter, receiver, and coupling lenses.

To demonstrate a possible real-world application of the prism, several terahertz communication links were established incorporating the prism, and some photographs of these experimental setups are shown in Fig. 5. These free-space communication demonstrations were done using a dedicated terahertz system^22^ that could generate a 0.3 THz carrier-frequency (design frequency of the prism) wave and digitally modulate it at data rates up to 20 Gbps via Binary-Phase-Shift-Keying (BPSK). Using this system, we performed Bit-Error-Rate (BER) measurements that quantitatively revealed the fidelity of the communication link and produced “eye diagrams” for a qualitative assessment. Figure 5(a) shows a communication link with a 0.5 m line-of-sight path between the prism and the receiver with a polythene lens to improve coupling. Figure 5(b) gives a closeup view of the prism and the horn antenna attached to the transmitter in this setup. Figure 5(c) shows another communication link with a 7.5 m reflection path, where the terahertz beam reflects off the finished (painted, untextured) hallway drywall. A closeup view showing the prism, transmitter, receiver, and coupling lenses in this setup is given in Fig. 5(d). In all these experiments, the prism plate-separation was 1.2 mm corresponding to a beam deflection of 14°. Once the data links were established, BER measurements were performed while systematically varying the transmitter power, path length, or the data-rate for a comprehensive study.

**Figure 6:**
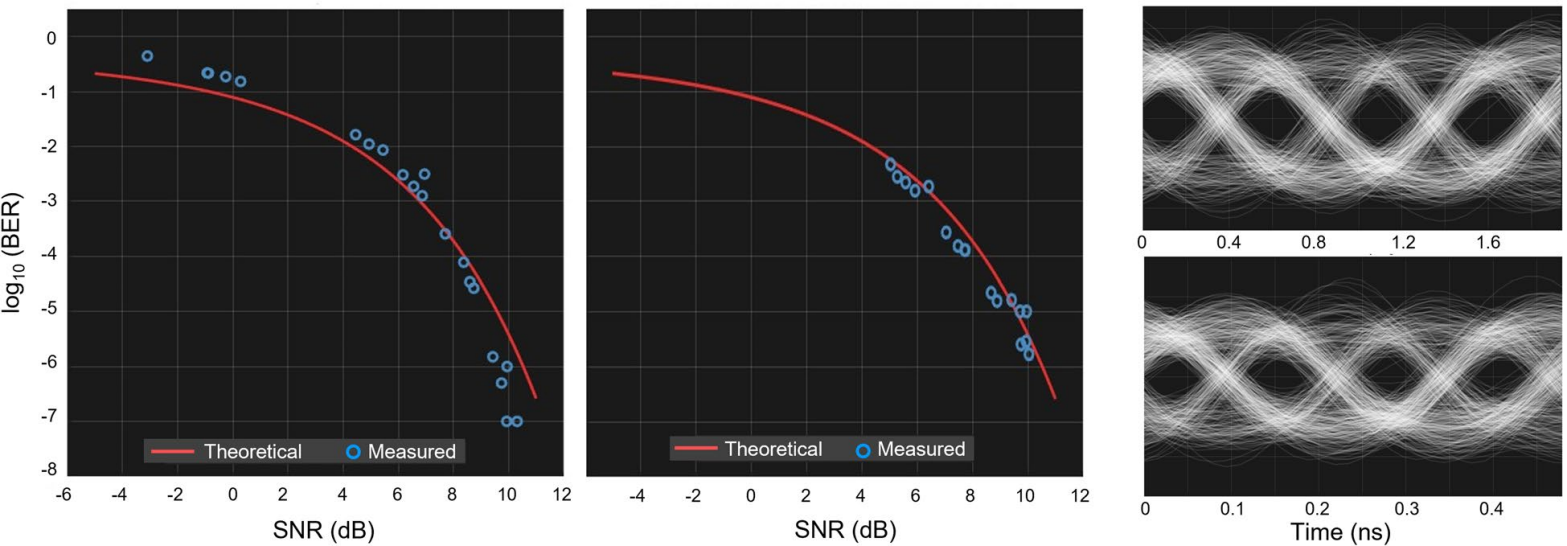
Results of the terahertz communication demonstrations. The left plot gives the BER versus SNR for a 0.5 m link with a 10 Gbps data-rate while varying the transmitter power. The right plot gives the BER versus the SNR for a 1.0 m link with a constant transmitter power while varying the data-rate from 1 to 19 Gbps. The eye diagrams correspond to 2 Gbps (upper) and 8 Gbps (lower) data rates.

Figure 6 presents the results of the data communication demonstrations that incorporated the prism. The left plot gives the measured BER versus SNR for a 0.5 m link with a 10 Gbps data-rate while varying the transmitter power. The right plot gives the measured BER versus the SNR for a 1.0 m link with a constant transmitter power while varying the data-rate from 1 to 19 Gbps. These measured values (blue circles) are compared to theoretical (red) curves based on well-established theory for free-space digital communication links^23^. The very good agreement between the measured and theoretical values imply that the prism does not impair the performance of the communication links. These plots reveal measured BER values down to 10^−7^, meaning that only one in every ten million bits of the input stream is in error in the demodulated data output, an excellent value for free-space communication. Furthermore, the representative eye diagrams shown in Fig. 6 correspond to data rates of 2 Gbps (upper panel) and 8 Gbps (lower panel) for the 1.0 m link. Here, the clear eye openings qualitatively indicate once again high-fidelity communication through the AD prism.

It is important to consider the allowable bandwidth of the device which is determined by the angular shift of the beam when the signal superimposes on the carrier frequency for a given plate separation. Assuming that the detector accepts a 4° half angle, we have theoretically estimated the range of this bandwidth for a 0.3 THz carrier. This transmittable bandwidth varies from 30 to 150 GHz, corresponding to a plate separation of 0.8 mm (with a nominal carrier deflection of 31°) and a plate separation of 2 mm (with a nominal carrier deflection of 5°), respectively. At a plate separation of 1.2 mm (with a nominal carrier deflection of 14°), that is when the prism was integrated into the communication channel, it can support a bandwidth of 70 GHz, well above what was necessary for the experimentally demonstrated maximum data rates.

## Conclusion

We experimentally demonstrated that a 0.3 THz beam can be steered through an angle of 29° using an AD prism, where this deflection angle is limited only by the precision of the spacers. These static-scanning test results prove that we can indeed scan a terahertz beam in space by compressing and expanding the plate assembly of the AD prism. To demonstrate possible dynamic scanning of the beam, we also carried out experiments with spring-loaded spacers, demonstrating continuous scanning of the beam in 1° steps. Furthermore, the measured input/output beam intensity maps revealed very good beam quality and an estimated overall power efficiency of 71% (1.5 dB insertion loss). Finally, to demonstrate its use in a real-world application, we integrated the prism into the path of a free-space terahertz communication link and demonstrated unimpaired performance. This demonstration highlights its potential use in future point-to-point 6G wireless communication systems that require scanned terahertz beams or pointing corrections, encompassing applications ranging from mobile receivers to backhaul links affected by jitter and antenna mast vibrations. In conclusion, this experimental investigation demonstrates another powerful avenue of terahertz wave control using ADs.

## Data Availability

The datasets generated during and/or analyzed during the current study are available from the corresponding author on reasonable request.
